# Comparative efficacy of green exercise versus indoor exercise for depression and anxiety: a systematic review and network meta-analysis

**DOI:** 10.3389/fpubh.2026.1831073

**Published:** 2026-05-28

**Authors:** Yun Liang, Da-Yuan Wei, Su Min

**Affiliations:** Department of Anesthesiology, The First Affiliated Hospital of Chongqing Medical University, Chongqing, China

**Keywords:** anxiety, depression, green exercise, indoor exercise, physical activity

## Abstract

**Background:**

Physical activity is a proven intervention for depression and anxiety, but the optimal environment for exercising remains debated. While “green exercise” (in nature) is theorized to offer superior mental health benefits to indoor exercise, a comprehensive quantitative ranking of these modalities against a no-exercise control is lacking. We aimed to determine the comparative efficacy of green exercise, indoor exercise, and control conditions for improving mental health outcomes.

**Methods:**

We conducted a systematic review and network meta-analysis of randomized controlled trials (RCTs). We searched PubMed, Embase, Web of Science, and CENTRAL from inception to December 2024 for RCTs comparing at least two of the following: green exercise, indoor exercise, or a no-exercise/standard care control group in adults. The primary outcome was a composite measure of mental health, including symptoms of depression, anxiety, and psychological distress. A random-effects network meta-analysis was performed to calculate standardized mean differences (SMDs) and rank the interventions using P-scores. The protocol was registered with PROSPERO (CRD420261279222).

**Results:**

Fifteen RCTs involving 809 participants were included. The network of evidence formed a closed three-node loop. Compared to control, green exercise showed a large and statistically significant effect on improving mental health (SMD = −0.96, 95% CI: −1.66 to −0.25). Indoor Exercise showed a small, non-significant beneficial trend (SMD = −0.21, 95% CI: −1.08 to 0.67). In direct comparison, green exercise was significantly superior to indoor exercise (SMD = −0.75, 95% CI: −1.32 to −0.18). Ranking analysis confirmed green exercise as the most effective intervention with near certainty (*p*-score = 0.99). The superior effect of green exercise was maintained in a subgroup analysis of clinical populations.

**Conclusion:**

Green exercise is a highly effective intervention for improving mental health, demonstrating benefits significantly greater than both indoor exercise and no-exercise control conditions. These findings strongly support the integration of nature-based physical activity into clinical practice and public health strategies for mental wellness.

**Systematic review registration:**

PROSPERO, CRD420261279222.

## Introduction

Depression is a leading cause of disability worldwide, affecting over 280 million people and representing a significant global public health challenge (30). While established treatments such as psychotherapy and pharmacotherapy are effective for many, a substantial number of individuals experience only partial remission, relapse, or face barriers to access, including cost, stigma, and side effects (1, 2). Consequently, there is a pressing need for accessible, scalable, and cost-effective complementary interventions to support mental health.

Physical activity has emerged as a powerful tool in both the prevention and treatment of depression (3, 4). A landmark meta-analysis demonstrated that exercise can be as effective as antidepressant medication or psychotherapy for mild to moderate depression (5). The antidepressant mechanisms of exercise are multifaceted, involving neurobiological pathways such as the promotion of neurogenesis, regulation of the hypothalamic–pituitary–adrenal (HPA) axis, and modulation of inflammatory processes (6, 7). Beyond its biological effects, exercise also confers psychological benefits, including enhanced self-efficacy, distraction from negative thoughts, and increased social interaction (8).

However, a critical yet often overlooked variable is the environment in which physical activity is performed. A growing body of research has begun to differentiate between “green exercise” (physical activity performed in natural environments) and “indoor exercise” (activity in synthetic or built settings). The theoretical foundation for the added benefit of green exercise is robust, drawing from concepts like the Biophilia Hypothesis, which posits an innate human affinity for nature (31). This is particularly relevant given that modern psychological distress is often exacerbated by social isolation, loneliness, and chronic stress—factors that have been identified as primary contributors to the global depression epidemic. In this context, the Japanese concept of ‘Shinrin-yoku’ (forest bathing) has gained international recognition, suggesting that immersive experiences in nature can significantly mitigate these stressors and promote emotional regulation, closely aligning with the biophilic principles mentioned above. Additionally, Attention Restoration Theory, which suggests natural settings can restore cognitive resources depleted by urban life (9).

Numerous studies have provided evidence supporting these theories. Systematic reviews and meta-analyses have consistently found that physical activity in natural environments is associated with greater improvements in self-esteem, mood, and perceived restorativeness compared to exercising indoors (10, 11). Even brief exposure to natural scenes has been shown to reduce stress and improve affective states more effectively than exposure to urban scenes (12, 13). Furthermore, individuals often report greater enjoyment and intention to repeat the activity when exercising outdoors, which has important implications for long-term adherence (14, 15).

Despite this accumulating evidence, the existing literature has a significant limitation: a scarcity of direct, quantitative comparisons between green exercise, indoor exercise, and no-exercise control groups within a unified analytical framework. Most meta-analyses have focused on pairwise comparisons (e.g., green vs. indoor), often without a non-active control, or have pooled heterogeneous control groups. This makes it difficult to ascertain whether the benefits of green exercise are simply additive or if there is a synergistic effect that makes it superior to both indoor exercise and no intervention. To date, no study has systematically synthesized both direct and indirect evidence to rank these three common strategies for managing depressive symptoms.

A network meta-analysis (NMA) is the ideal methodology to address this gap. NMA allows for the simultaneous comparison of multiple interventions within a single model, preserving the randomized nature of the included trials while incorporating both direct (from head-to-head trials) and indirect evidence (16, 17). By applying this powerful statistical approach, we can move beyond simple pairwise comparisons to estimate the relative efficacy of green exercise, indoor exercise, and control conditions and generate a clinically meaningful hierarchy of these interventions.

Therefore, the objective of this systematic review and network meta-analysis was to determine the comparative efficacy of green exercise versus indoor exercise for improving depression and related mental health outcomes, using a no-exercise or standard care control as a common comparator. We hypothesized that green exercise would be the most effective intervention, followed by indoor exercise, and that both would be superior to the control condition.

## Methods

### Protocol and registration

This systematic review and network meta-analysis was conducted and reported in accordance with the Preferred Reporting Items for Systematic Reviews and Meta-Analyses (PRISMA) extension statement for network meta-analyses (PRISMA-NMA) (17). The protocol for this review was registered in the International Prospective Register of Systematic Reviews (PROSPERO) under the registration number [CRD420261279222].

### Eligibility criteria

Studies were included based on the following PICOS (Population, Intervention, Comparator, Outcomes, Study design) criteria:

Population (P): studies involving adult participants (aged 18 years or older) from either clinical populations (e.g., with diagnosed depression, anxiety, or other mental health conditions) or non-symptomatic populations (e.g., university students, general community).

Intervention (I): green exercise, defined as any form of physical activity (e.g., walking, running, cycling, calisthenics) performed in a real natural outdoor environment (e.g., parks, forests, beaches, nature trails).

Comparators (C): studies had to include at least one of the following comparator groups:

Indoor exercise: the same or a comparable physical activity performed in an indoor or built environment (e.g., laboratory treadmill, fitness center, urban streets).

Control: a no-exercise control group, which could be a wait-list control, standard care, or a sedentary active control (e.g., sitting, occupational therapy).

Outcomes (O): studies had to report at least one quantitative psychological outcome related to mental health. The primary outcomes of interest were symptoms of depression, anxiety, or general psychological distress. Secondary outcomes included positive affect, vitality, and mental well-being.

Study design (S): only randomized controlled trials (RCTs), including both parallel-group and crossover designs, were eligible for inclusion.

Studies involving virtual reality (VR) or simulated nature environments were excluded to maintain the focus on “real” environmental effects.

#### Information sources and search strategy

A comprehensive systematic search was conducted in the following electronic databases from their inception to December 2025: PubMed, Embase, Web of Science, and the Cochrane Central Register of Controlled Trials (CENTRAL). The search strategy combined keywords and subject headings related to three core concepts: (1) the population/outcome (e.g., “depression,” “anxiety,” “mental health”), (2) the interventions (e.g., “green exercise,” “outdoor activity,” “nature,” “indoor exercise,” “treadmill”), and (3) the study design (e.g., “randomized controlled trial”). The full search strategy for PubMed is provided in Supplementary Appendix S1. Additionally, the reference lists of included studies and relevant systematic reviews were manually screened to identify any further eligible trials (snowballing).

### Study selection and data extraction

Two reviewers (YL and DW) independently screened titles and abstracts for eligibility. Full-text articles of potentially relevant studies were then retrieved and assessed against the inclusion criteria. Any disagreements were resolved through discussion or by consulting a third reviewer (MS).

A standardized data extraction form was developed in Microsoft Excel. The same two reviewers independently extracted the following data from each included study: first author and year of publication, country, study design, participant characteristics (sample size, age, sex, clinical status), details of the Green, Indoor, and Control interventions (type, duration, frequency, intensity), outcome measures, and results for each outcome (mean, standard deviation, and sample size for each group at post-intervention or change-from-baseline scores).

### Risk of bias assessment

The methodological quality of the included RCTs was independently assessed by two reviewers using the Cochrane Risk of Bias tool 2 (RoB 2) for randomized trials (18). The tool assesses bias across five domains: (1) bias arising from the randomization process, (2) bias due to deviations from intended interventions, (3) bias due to missing outcome data, (4) bias in measurement of the outcome, and (5) bias in selection of the reported result. Each domain was judged as “Low risk,” “Some concerns,” or “High risk” of bias. An overall risk of bias was then determined for each study.

### Data synthesis and analysis

A random-effects network meta-analysis was performed using the netmeta package in R (Version 4.2.0) (19). For each study, the standardized mean difference (SMD) and its standard error were calculated for all available pairwise comparisons, as different scales were used to measure mental health outcomes across studies. For outcomes where a higher score indicated better mental health (e.g., vitality, well-being), the mean values were multiplied by −1 to ensure all SMDs shared a consistent direction, where a negative SMD represents a favorable outcome.

The primary analysis was a three-node model comparing green exercise, indoor exercise, and control. We estimated the summary SMD and 95% confidence intervals (CIs) for each intervention relative to the control group. The relative ranking of the three interventions was evaluated using *p*-scores, which represent the probability that an intervention is the best among those being compared (19).

Consistency between direct and indirect evidence in the network was assessed using the node-splitting method. Statistical heterogeneity was quantified using the *I*^2^ statistic. Subgroup analyses were planned for symptomatic/non-symptomatic populations and for negative vs. positive psychological outcomes. Publication bias was visually assessed using a contour-enhanced funnel plot. The significance level was set at *p* < 0.05 for all analyses.

## Results

### Study selection

**Figure 1:**
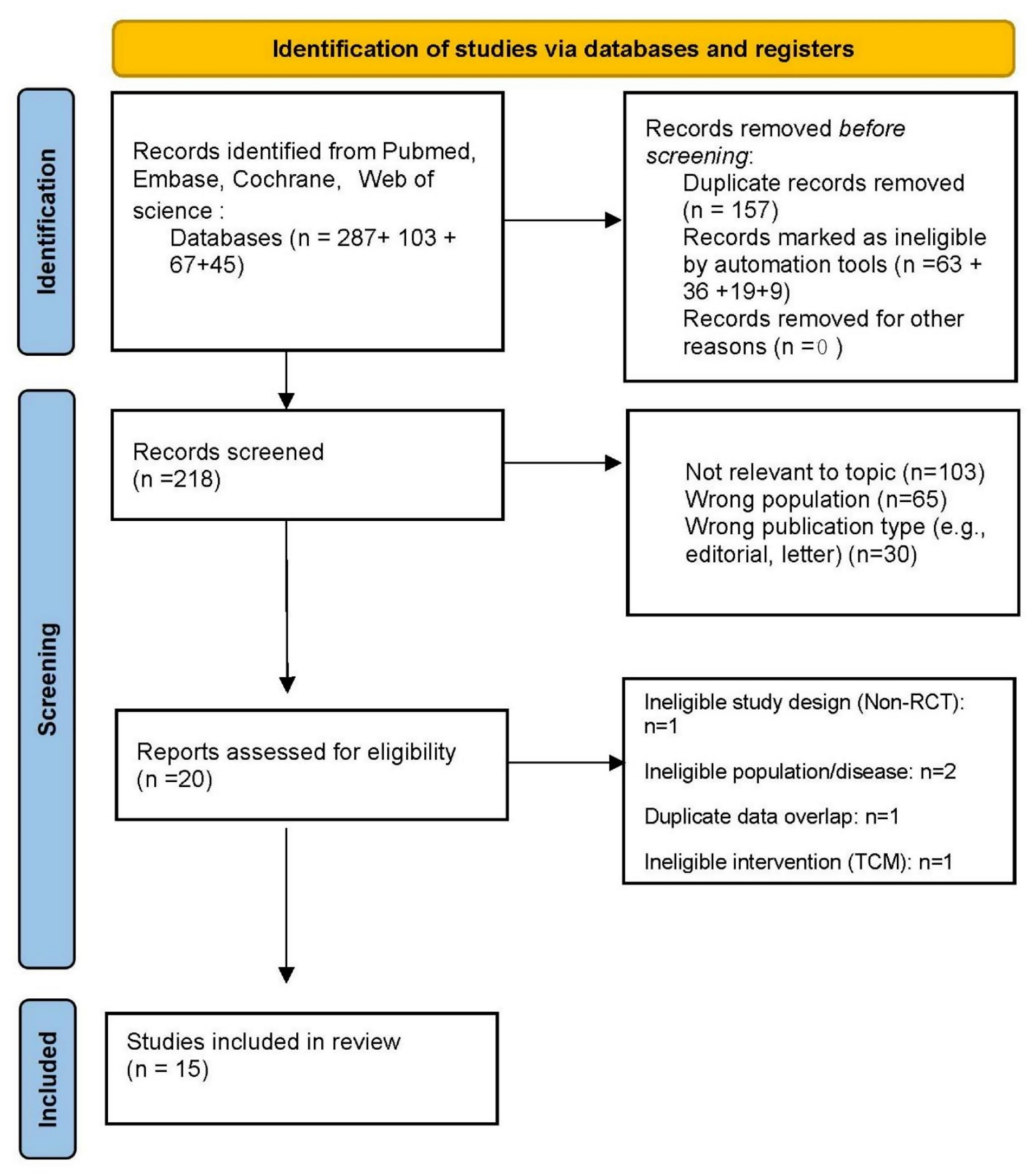
PRISMA flow diagram of the study selection process. The flowchart details the number of records identified, screened, assessed for eligibility, and included in the final qualitative synthesis and network meta-analysis (*N* = 15 studies).

The systematic search initially identified 455 records from electronic databases. After removing duplicates and screening titles and abstracts, 20 full-text articles were assessed for eligibility. Of these, 5 were excluded due to ineligible study design, population, or intervention type. Finally, 15 randomized controlled trials (RCTs) meeting the inclusion criteria were included in the systematic review and network meta-analysis (Figure 1). The characteristics of these studies are summarized in Table 1.

**Table 1:** League table of the network meta-analysis results for global mental health.

Intervention	Control	Green	Indoor
Control	Control		
Green	SMD = **−0.96** (CI: −0.25 to −1.66)	Green	
Indoor	SMD = **−0.21** (CI: 0.67 to −1.08)	SMD = **−0.75** (CI: −1.32 to −0.18)	Indoor

Values represent the standardized mean difference (SMD) and 95% confidence intervals (in parentheses) for the row treatment compared with the column treatment. Bold values indicate statistical significance. Negative SMDs favor the row treatment (better outcome).

Green −0.75 (−1.32 to −0.18), indoor −0.96 (−1.66 to −0.25) −0.21 (−1.08 to 0.67) Control.

### Network structure and characteristics

**Figure 2:**
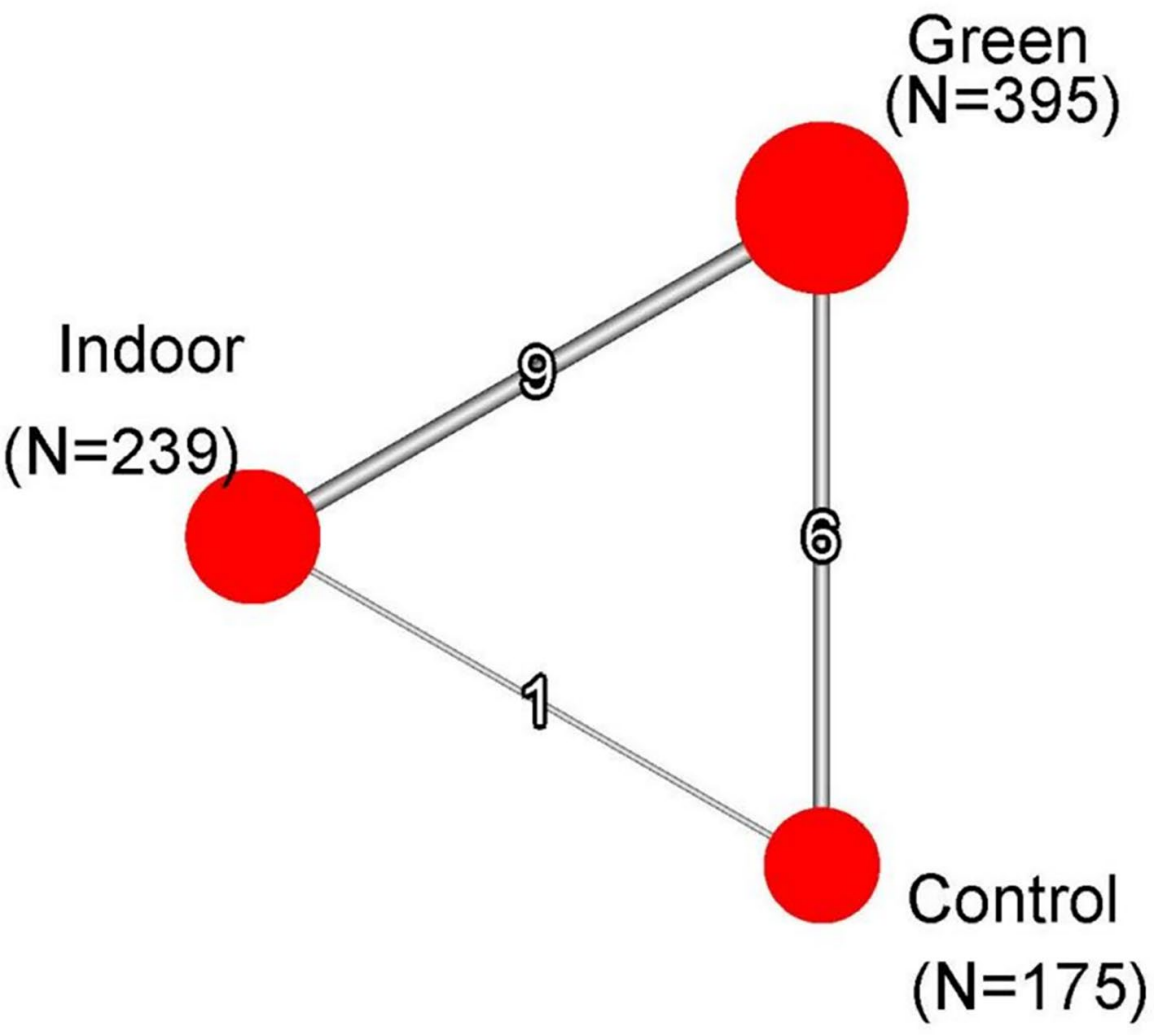
Network plot of comparisons for global mental health. The nodes (circles) represent the intervention conditions (green: green exercise; indoor: indoor exercise; Control: no intervention/usual care). The size of the nodes is proportional to the number of participants allocated to each group (green *n* = 395; indoor *n* = 239; control *n* = 175). The thickness of the connecting lines is proportional to the number of studies directly comparing the two conditions. The numbers on the lines indicate the count of studies for each pairwise comparison.

The network of evidence for global mental health outcomes is illustrated in Figure 2. The analysis included a total of 809 participants across 15 studies. The network geometry formed a closed loop, enabling both direct and indirect comparisons among three intervention nodes: green exercise (*n* = 395 participants), indoor exercise (*n* = 239 participants), and control (*n* = 175 participants). The direct comparison between green and indoor exercise was supported by the largest body of evidence (9 studies), followed by Green versus control (6 studies). A single multi-arm trial (32) provided the only direct evidence linking indoor exercise to the control condition, thereby closing the network loop.

### Risk of bias assessment

The methodological quality of the included studies was moderate (Supplementary Figures S1, S2). While most studies demonstrated a low risk of bias in the randomization process, biases arising from deviations from the intended intervention were common due to the inability to blind participants to exercise conditions. Several studies were judged to be at a high risk of bias due to significant participant attrition, notably Brown et al. (33), Bang et al. (34), and Ryu et al. (35).

### Primary outcome: global mental health

**Figure 3:**
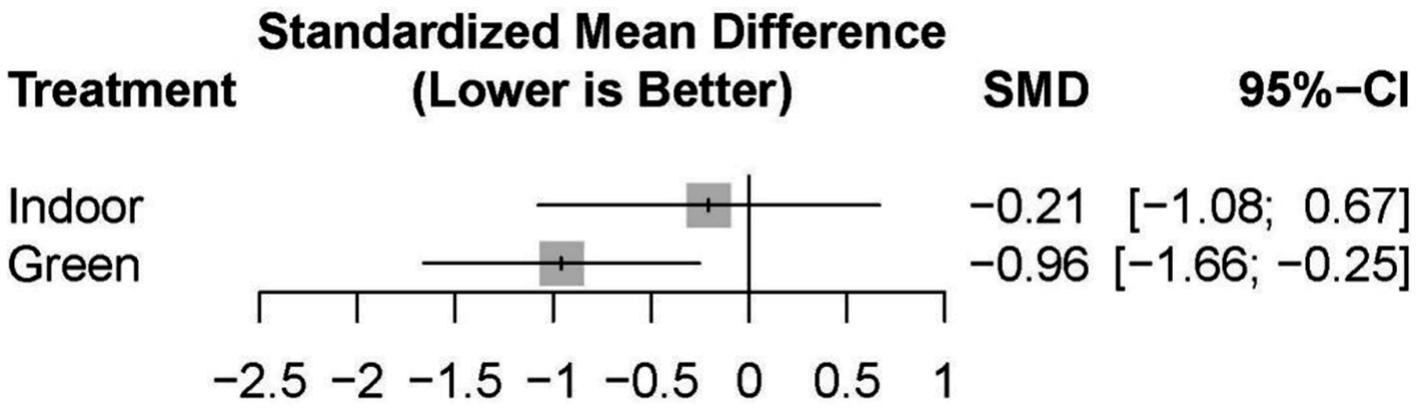
Forest plot of relative effects of exercise interventions compared with control on global mental health. Data are expressed as standardized mean differences (SMD) with 95% confidence intervals (CI). A random-effects model was used. SMDs less than 0 indicate a beneficial effect (favoring the intervention) in reducing negative mental health symptoms or increasing positive well-being (inverted). Green: SMD −0.96 [−1.66; −0.25]. Indoor: SMD −0.21 [−1.08; 0.67].

The results of the network meta-analysis for global mental health outcomes are presented in Figure 3 (Forest Plot) and Table 1 (League Table).

Compared with the control group (no intervention/standard care), green exercise demonstrated a large and statistically significant effect in improving mental health outcomes [standardized mean difference (SMD) = −0.96, 95% CI: −1.66 to −0.25]. Indoor exercise also showed a trend towards improvement, but the effect was small and not statistically significant (SMD = −0.21, 95% CI: −1.08 to 0.67).

In the head-to-head comparison derived from the league table (Table 1), green exercise was found to be statistically superior to indoor exercise (SMD = −0.75, 95% CI: −1.32 to −0.18).

Consistent with these findings, the *p*-score ranking probabilities confirmed that green exercise had the highest probability of ranking as the most effective intervention (*p*-score = 0.99), far exceeding both indoor exercise (*p*-score = 0.34) and control (*p*-score = 0.16) (Supplementary Figure S3).

### Subgroup analysis: symptomatic populations

To assess the robustness of these findings in clinical settings, a subgroup analysis was conducted on six trials that recruited participants with diagnosed clinical conditions, including major depressive disorder (36, 37, 38), schizophrenia (35), multiple sclerosis (38), and frailty in older adults (39, 40).

**Figure 4:**
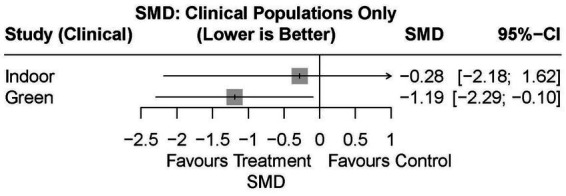
Subgroup analysis of relative effects in clinical populations. Forest plot showing the SMDs of green and indoor exercise compared to control, specifically in participants with diagnosed clinical conditions (e.g., depression, schizophrenia). Green exercise shows a significant large effect (SMD = −1.19), whereas indoor exercise does not differ significantly from control.

As shown in Figure 4, the superior efficacy of green exercise was maintained and slightly amplified in these symptomatic populations (SMD = −1.19, 95% CI: −2.29 to −0.10) when compared to control. In contrast, indoor exercise did not show a significant benefit over control in this subgroup (SMD = −0.28, 95% CI: −2.18 to 1.62).

### Subgroup analysis by outcome type

When outcomes were stratified into negative and positive domains, green exercise demonstrated a large and significant effect on reducing negative affect (e.g., depression, anxiety) (SMD = −1.20, 95% CI: −2.07 to −0.32) (Supplementary Figure S4). For positive well-being outcomes (e.g., vitality, quality of life), green exercise also showed a favorable trend, although the effect was not statistically significant (SMD = −0.48, 95% CI: −1.08 to 0.13) (Supplementary Figure S5).

### Publication bias and inconsistency

The contour-enhanced funnel plot (Supplementary Figure S6) was largely symmetrical, suggesting no strong evidence of publication bias. The node-splitting analysis revealed no significant inconsistency between direct and indirect evidence for any of the comparisons (all *p* > 0.05), indicating that the network model was consistent and robust.

## Discussion

### Principal findings

This network meta-analysis, synthesizing data from 15 randomized controlled trials, provides robust quantitative evidence that green exercise is a highly effective intervention for improving mental health outcomes. Our primary finding is twofold: not only is exercising in natural environments significantly superior to no-intervention control conditions (SMD −0.96), but it is also statistically more effective than exercising in indoor or built environments (SMD vs. Indoor: −0.75). In contrast, while indoor exercise showed a beneficial trend, its effect was not statistically distinguishable from the control group in our main analysis. Crucially, the superior efficacy of green exercise was maintained, and even amplified, within the subgroup of symptomatic populations, highlighting its potential as a therapeutic tool.

### Comparison with existing literature

Our results confirm and extend the findings of previous systematic reviews that have consistently reported the psychological benefits of nature-based physical activity (10, 11). While earlier meta-analyses established that green exercise was generally more beneficial than indoor alternatives, our network meta-analysis provides a more precise, consolidated estimate of this superiority. A recent meta-analysis also found stronger immediate psychological benefits for outdoor physical activity in natural versus urban environments, though it did not include a no-exercise control, underscoring the unique contribution of our three-node network (20). The large effect size observed for green exercise aligns with broader epidemiological evidence linking greenspace exposure to reduced risks of depression and anxiety (21). Our finding that indoor exercise was not significantly more effective than control is noteworthy and suggests that the context of physical activity is a critical determinant of its mental health benefits.

### Potential mechanisms

The marked superiority of green exercise can be interpreted through several complementary theoretical frameworks. The Attention Restoration Theory (ART) posits that natural environments, rich in soft fascinations, allow for the restoration of directed attention, thereby reducing mental fatigue (9). Simultaneously, the Stress Reduction Theory (SRT) suggests that exposure to unthreatening natural settings triggers a rapid, positive affective response and reduces physiological arousal (13). These theories are further supported by the Biophilia Hypothesis, which proposes an innate human tendency to connect with nature (31). Beyond these psychological mechanisms, exercising outdoors provides multisensory stimulation (e.g., sunlight, fresh air, natural sounds) that is absent in sterile indoor environments. Physiologically, this may translate to more favorable changes in autonomic nervous system activity and stress hormone profiles (12). The combination of these restorative properties with the known benefits of physical activity likely creates a synergistic effect, amplifying the overall positive impact on mental health (22).

Substantial clinical and methodological heterogeneity was observed across the included studies, particularly regarding intervention duration (ranging from single bouts to several months) and the specific types of green exercise. While our subgroup analysis of symptomatic populations confirmed the robustness of green exercise, future research should utilize meta-regression to more precisely determine how exercise intensity and baseline symptom severity influence the overall effect size.

### Strengths and limitations

The primary strength of this study is its use of the network meta-analysis methodology, which, to our knowledge, is the first to be applied to this specific question. This approach allowed for the simultaneous comparison of three distinct conditions (Green, Indoor, Control) and the estimation of relative effects, even for comparisons with limited direct evidence, adhering to PRISMA-NMA guidelines (17). The power of the NMA method has been demonstrated in landmark psychiatric studies (16), and our application here provides a new level of clarity to the field. Further strengths include the inclusion of the most recent evidence, with several trials from 2024 and 2025, and a robust assessment of network consistency which revealed no significant discrepancies between direct and indirect evidence.

However, several limitations must be acknowledged. First, there was considerable heterogeneity among the included studies in terms of population (from healthy students to patients with schizophrenia), intervention duration (from single bouts to 6-month programs), and specific type of exercise. Second, our “Indoor” node combined various settings, including laboratory treadmills, fitness centers, and urban streets. While this grouping is pragmatically necessary for a connected network, it may obscure nuanced differences between various types of non-natural environments. Third, most included trials focused on acute or short-term effects, limiting our ability to draw conclusions about the long-term sustainability of these benefits or adherence to the interventions. Finally, as with all exercise research, blinding of participants was not feasible, introducing a potential risk of performance bias, which was noted in our risk of bias assessment.

However, the high ranking of green exercise (*p*-score = 0.99) should be interpreted with caution. While our findings suggest near-certain superiority, *p*-scores reflect relative ranking probabilities based on point estimates and do not fully account for the magnitude of clinical differences or the certainty of evidence. Given the relatively small number of included RCTs and the sparse nature of certain nodes in the network (specifically the single link between indoor exercise and control), there remains a risk of overestimating the effect size.

Another critical limitation is the aggregation of heterogeneous settings—such as laboratory treadmills, indoor gyms, and built urban environments—into a single ‘Indoor Exercise’ node. This pragmatic grouping was necessary for network connectivity but may obscure nuanced differences between varied non-natural settings, potentially affecting the interpretation of the comparative hierarchy.

Furthermore, the network structure was relatively sparse, with only a single multi-arm trial providing a direct link between indoor exercise and the no-exercise control. This imbalance in evidence loops raises concerns regarding the stability of indirect comparisons, suggesting that the estimated relative efficacy between indoor and outdoor exercise should be considered exploratory until more direct head-to-head trials are available.

### Implications for clinical practice and public health

Our findings have significant implications. For clinicians and psychotherapists, “green prescriptions” or recommending outdoor physical activity should be considered a potent, low-cost, and accessible adjunct therapy for individuals with depression and anxiety, a conclusion supported by our strong findings in clinical populations. This aligns with a growing body of evidence supporting nature-based interventions for mental health (4, 23). For public health policy, our results underscore the critical importance of creating, maintaining, and ensuring equitable access to high-quality urban green and blue spaces (24, 25). The evidence suggests that such access is not merely an aesthetic amenity but a vital component of public health infrastructure that can mitigate mental health burdens (26).

### Future research directions

Beyond immediate clinical efficacy, the long-term adherence and sustainability of nature-based interventions remain vital for public health translation. Future policies must also address equity and access to high-quality green spaces, ensuring that socio-economically disadvantaged populations—who often face the highest burden of mental health issues—can benefit from ‘green prescriptions’ without systemic barriers to natural environments.

Future research should focus on several key areas. Large-scale, long-term RCTs are needed to determine the sustainability of mental health benefits and patterns of adherence. Studies investigating the optimal “dose” of green exercise—in terms of frequency, duration, and intensity—are crucial for developing clear clinical guidelines (27, 28). Furthermore, research employing neurobiological measures could help elucidate the underlying mechanisms, for instance by examining changes in hippocampal volume or inflammatory markers (29). Finally, head-to-head trials comparing different types of natural environments (e.g., forests vs. beaches) could provide more nuanced recommendations.

## Conclusion

In conclusion, this network meta-analysis provides the most comprehensive quantitative evidence to date that exercising in natural environments is a superior strategy for improving mental health compared to both exercising indoors and no intervention. The benefits are substantial, statistically significant, and extend to clinical populations. These findings strongly advocate for the integration of green exercise into mental health treatment plans and public health promotion strategies.

## Data Availability

The original contributions presented in the study are included in the article/Supplementary material, further inquiries can be directed to the corresponding author.

## References

[1] Cuijpers P Noma H Karyotaki E Vink T Cipriani A. A network meta-analysis of the effects of psychotherapies, pharmacotherapies and their combination in the treatment of adult depression. World Psychiatry. (2020) 19:92–107. doi: 10.1002/wps.20701 31922679 PMC6953550

[2] Rush AJ Trivedi MH Wisniewski SR Nierenberg AA Stewart JW Warden D . Acute and longer-term outcomes in depressed outpatients requiring one or several treatment steps: a STAR*D report. Am J Psychiatry. (2006) 163:1905–17. doi: 10.1176/ajp.2006.163.11.1905 17074942

[3] Schuch FB Vancampfort D Richards J Rosenbaum S Ward PB Stubbs B. Exercise as a treatment for depression: a meta-analysis of 35 RCTs. Am J Psychiatry. (2018) 173:1011–21. doi: 10.1176/appi.ajp.2016.15111364

[4] Singh B Olds T Curtis R Dumuid D Virgara R Watson A . Effectiveness of physical activity interventions for improving depression, anxiety and distress: an overview of systematic reviews. Br J Sports Med. (2023) 57:1203–9. doi: 10.1136/bjsports-2022-106195, 36796860 PMC10579187

[5] Kandola A Vancampfort D Herring M Rebar A Hallgren M Firth J . Moving to beat depression: a systematic review and meta-analysis of the effectiveness of exercise for major depressive disorder. Sports Med. (2019) 49:1503–26. doi: 10.1007/s40279-019-01124-6

[6] Ernst C Olson AK Pinel JP Lam RW Christie BR. Antidepressant effects of exercise: evidence for an adult-neurogenesis hypothesis? J Psychiatry Neurosci. (2006) 31:84–92. doi: 10.1139/jpn.0610, 16575423 PMC1413959

[7] Meeusen R De Meirleir K. Exercise and brain neurotransmission. Sports Med. (1995) 20:160–88. doi: 10.2165/00007256-199520030-00004, 8571000

[8] Craft LL Perna FM. The benefits of exercise for the clinically depressed. Prim Care Companion J Clin Psychiatry. (2004) 6:104. doi: 10.4088/pcc.v06n0301, 15361924 PMC474733

[9] Kaplan S. The restorative benefits of nature: toward an integrative framework. J Environ Psychol. (1995) 15:169–82. doi: 10.1016/0272-4944(95)90001-2

[10] Lahart I Darcy P Gidlow C Calogiuri G. The effects of green exercise on physical and mental wellbeing: a systematic review. Int J Environ Res Public Health. (2019) 16:1352. doi: 10.3390/ijerph16081352, 30991724 PMC6518264

[11] Thompson Coon J Boddy K Stein K Whear R Barton J Depledge MH. Does participating in physical activity in outdoor natural environments have a greater effect on physical and mental wellbeing than physical activity indoors? A systematic review. Environ Sci Technol. (2011) 45:1761–72. doi: 10.1021/es102947t, 21291246

[12] Gladwell VF Brown DK Wood C Sandercock GR Barton JL. The great outdoors: how a green exercise environment can benefit all. Extrem Physiol Med. (2013) 2:3. doi: 10.1186/2046-7648-2-3, 23849478 PMC3710158

[13] Ulrich RS. "Aesthetic and affective response to natural environment". In: Behavior and the Natural Environment. Boston: Springer (1983). p. 85–125.

[14] Calogiuri G Chroni S. The impact of the natural environment on the promotion of active living: an integrative systematic review. BMC Public Health. (2014) 14:873. doi: 10.1186/1471-2458-14-873, 25150711 PMC4246567

[15] Focht BC. Brief walks in outdoor and laboratory environments: effects on affective responses, enjoyment, and intentions to walk for exercise. Res Q Exerc Sport. (2009) 80:611–20. doi: 10.1080/02701367.2009.10599600, 19791648

[16] Cipriani A Furukawa TA Salanti G Chaimani A Atkinson LZ Ogawa Y . Comparative efficacy and acceptability of 21 antidepressant drugs for the acute treatment of adults with major depressive disorder: a systematic review and network meta-analysis. Lancet. (2018) 391:1357–66. doi: 10.1016/S0140-6736(17)32802-7 29477251 PMC5889788

[17] Hutton B Salanti G Caldwell DM Chaimani A Schmid CH Altman DG . The PRISMA extension statement for reporting of systematic reviews incorporating network meta-analyses of health care interventions: checklist and explanations. Ann Intern Med. (2015) 162:777–84. doi: 10.7326/M14-2385, 26030634

[18] Sterne JA Savović J Page MJ Elbers RG Blencowe NS Boutron I . RoB 2: a revised tool for assessing risk of bias in randomised trials. BMJ. (2019) 366:s. doi: 10.1136/bmj.l4898, 31462531

[19] Rücker G Schwarzer G. Ranking treatments in frequentist network meta-analysis works well for outcomes with low heterogeneity. BMC Med Res Methodol. (2015) 15:5. doi: 10.1186/s12874-015-0060-8 26227148 PMC4521472

[20] Wicks C Barton J Orbell S Andrews L. Psychological benefits of outdoor physical activity in natural versus urban environments: a systematic review and meta-analysis of experimental studies. Appl Psychol Health Well Being. (2022) 14:1037–61. doi: 10.1111/aphw.12353, 35259287 PMC9544808

[21] Twohig-Bennett C Jones A. The health benefits of the great outdoors: a systematic review and meta-analysis of greenspace exposure and health outcomes. Environ Res. (2018) 166:628–37. doi: 10.1016/j.envres.2018.06.030, 29982151 PMC6562165

[22] Bratman GN Anderson CB Berman MG Cochran B de Vries S Flanders J . Nature and mental health: an ecosystem service perspective. Sci Adv. (2019) 5:eaax0903. doi: 10.1126/sciadv.aax0903, 31355340 PMC6656547

[23] Coventry PA Brown JE Pervin J Brabyn S Pateman R Gomm D. Nature-based outdoor activities for mental and physical health: systematic review and meta-analysis. SSM Popul Health. (2021) 16:100934. doi: 10.1016/j.ssmph.2021.100934 34646931 PMC8498096

[24] Lawton E Brymer E Clough P Denovan A. The relationship between the physical activity environment, nature relatedness, anxiety, and the psychological wellbeing of a group of regular exercisers. Front Psychol. (2017) 8:1058. doi: 10.3389/fpsyg.2017.01058 28694788 PMC5483473

[25] Van den Bosch M Sang ÅO. Urban natural environments as nature-based solutions for improved public health—a systematic review of reviews. Environ Res. (2017) 158:373–84. doi: 10.1016/j.envres.2017.05.040 28686952

[26] Mitchell R Popham F. Effect of exposure to natural environment on health inequalities: an observational population study. Lancet. (2008) 372:1655–60. doi: 10.1016/s0140-6736(08)61689-x, 18994663

[27] Barton J Pretty J. What is the best dose of nature and green exercise for improving mental health? A multi-study analysis. Environ Sci Technol. (2010) 44:3947–55. doi: 10.1021/es903183r, 20337470

[28] White MP Alcock I Grellier J Wheeler BW Hartig T Warber SL . Spending at least 120 minutes a week in nature is associated with good health and wellbeing. Sci Rep. (2019) 9:7730. doi: 10.1038/s41598-019-44097-3, 31197192 PMC6565732

[29] Firth J Solmi M Wootton RE Vancampfort D Schuch FB Hoare E . A meta-review of “lifestyle psychiatry”: the role of exercise, diet, and sleep in the prevention and treatment of mental disorders. World Psychiatry. (2018) 19:360–80. doi: 10.1002/wps.20773 PMC7491615 32931092

[30] World Health Organization. Depression and other common mental Disorders: Global Health Estimates. Geneva: World Health Organization (2021).

[31] Wilson EO. Biophilia. Cambridge, MA: Harvard University Press (1984).

[32] Harte JL Eifert GH Smith R. The effects of running and meditation on beta-endorphin, corticotropin-releasing hormone and cortisol in plasma, and on mood. Biol Psychol. (1995) 40:251–65. 7669835 10.1016/0301-0511(95)05118-t

[33] Brown DK Barton JL Pretty J Gladwell VF. Walks4Work: assessing the role of the natural environment in a workplace physical activity intervention. Scand J Work Environ Health. (2014) 40:390–9. 24623515 10.5271/sjweh.3421

[34] Bang KS Lee IS Kim SJ Song MK Park SE. The effects of urban forest-walking program on health promotion behavior, physical health, depression, and quality of life: a randomized controlled trial of office-workers. J Korean Acad Nurs. (2016) 46:140–8. 26963423 10.4040/jkan.2016.46.1.140

[35] Ryu J Jung JH Kim J Kim CH Lee HB Kim DH . Outdoor cycling improves clinical symptoms, cognition and objectively measured physical activity in patients with schizophrenia: a randomized controlled trial. J Psychiatr Res. (2020) 120:144–53. doi: 10.1016/j.jpsychires.2019.10.015 31678749

[36] Watkins-Martin K Bolanis D Richard-Devantoy S Pennestri M-H Malboeuf-Hurtubise C Philippe F . The effects of walking in nature on negative and positive affect in adult psychiatric outpatients with major depressive disorder: a randomized-controlled study. J Affect Disord. (2022) 318:291–8. doi: 10.1016/j.jad.2022.08.121 36058362

[37] Dickmeyer A Smith JJ Halpin S McMullen S Drew R Morgan P . Walk-and-talk therapy versus conventional indoor therapy for men with low mood: a randomised pilot study. Clin Psychol Psychother. (2025) 32:e70035. doi: 10.1002/cpp.70035 39907105 PMC11795730

[38] Hvid LG Steenberg JL Roy F Skovgaard L. Outdoor walking exercise therapy improves walking capacity and well-being in persons with multiple sclerosis: a randomized controlled trial. Ann Phys Rehabil Med. (2025) 68:101985. doi: 10.1016/j.rehab.2025.101985 40252300

[39] Lee JLC Wong AYL Ng PHF Fu SN Fong KNK Cheng ASK . Outdoor exercise facility-based integrative Mobile health intervention to support physical activity, mental well-being, and exercise self-efficacy among older adults with Prefrailty and frailty in Hong Kong: pilot feasibility randomized controlled trial study. JMIR Mhealth Uhealth. (2025) 13:e69259. doi: 10.2196/69259 40471669 PMC12179572

[40] Rantanen T Äyräväinen I Eronen J Lyyra T Törmäkangas T Vaarama M . The effect of an outdoor activities’ intervention delivered by older volunteers on the quality of life of older people with severe mobility limitations: a randomized controlled trial. Aging Clin Exp Res. (2015) 27:161–9. doi: 10.1007/s40520-014-0254-7 24952472

