# Supplementary material for: Comparative efficacy of green exercise versus indoor exercise for depression and anxiety: a systematic review and network meta-analysis
Source: Front Public Health. 2026 May 28;14:1831073. doi: 10.3389/fpubh.2026.1831073 (PMC13253268; doi:10.3389/fpubh.2026.1831073)
Supplement: Supplementary file 1 [file Supplementary_file_1.docx]

Supplementary Materials


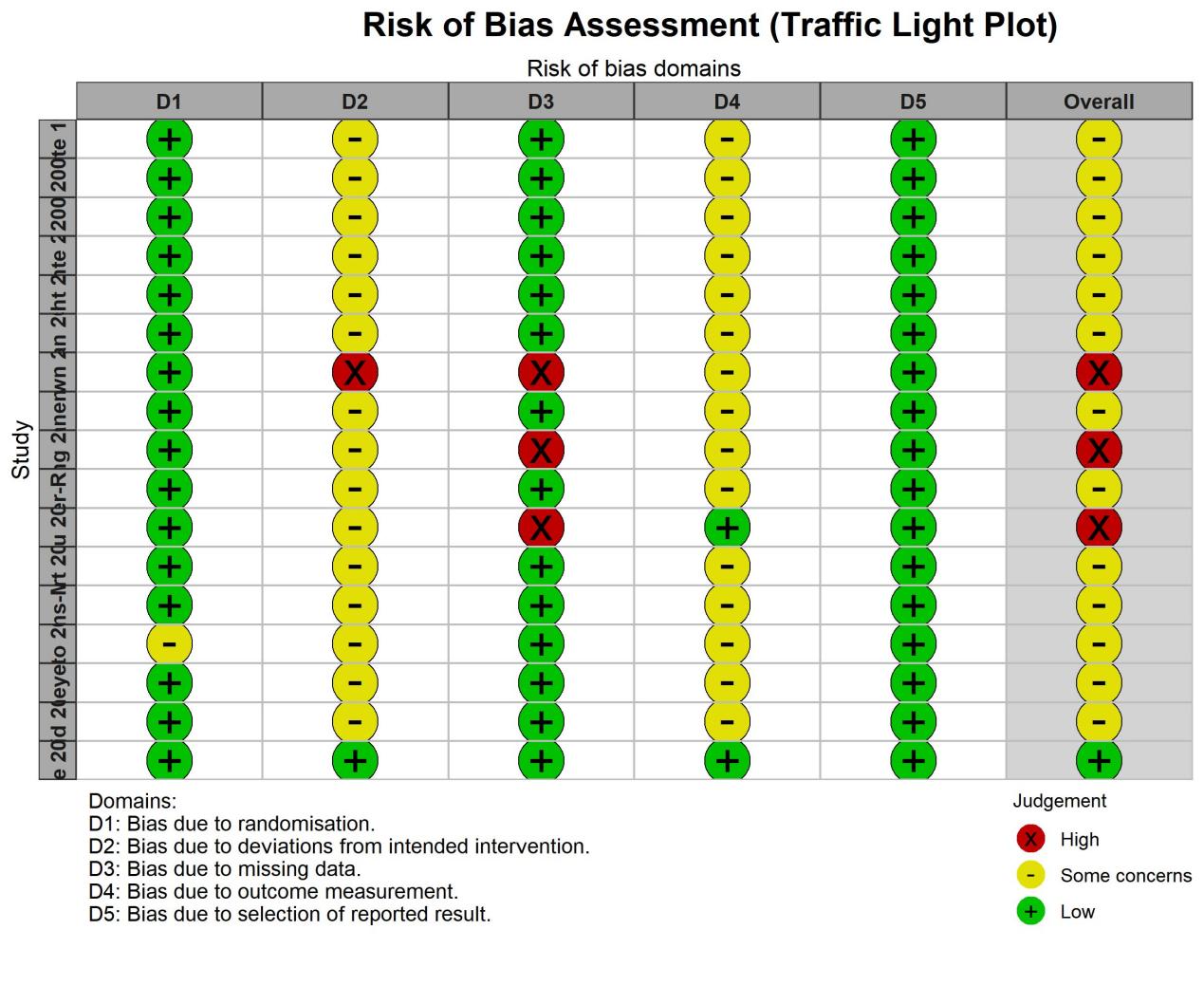
Figure S1. Risk of bias assessment for individual studies (Traffic Light Plot). Evaluation of bias domains using the Cochrane Risk of Bias tool (RoB 2.0). Green (+) indicates low risk, yellow (-) indicates some concerns, and red (x) indicates high risk.


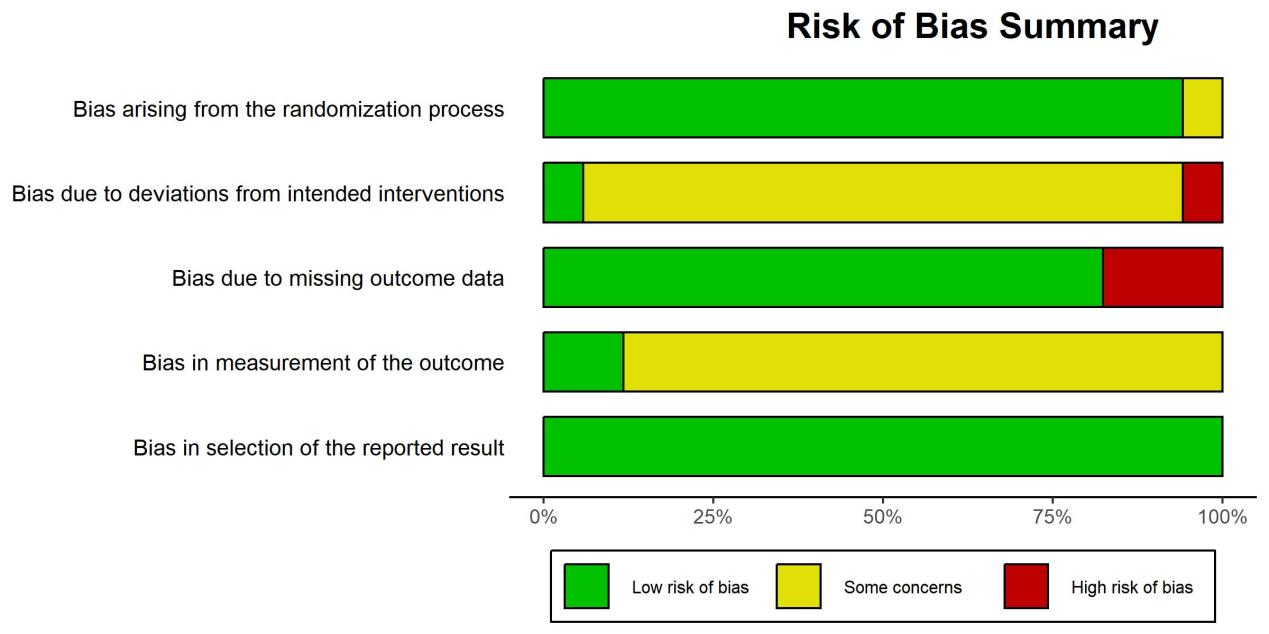
Figure S2. Risk of bias summary. Review authors' judgments about each risk of bias item presented as percentages across all included studies.


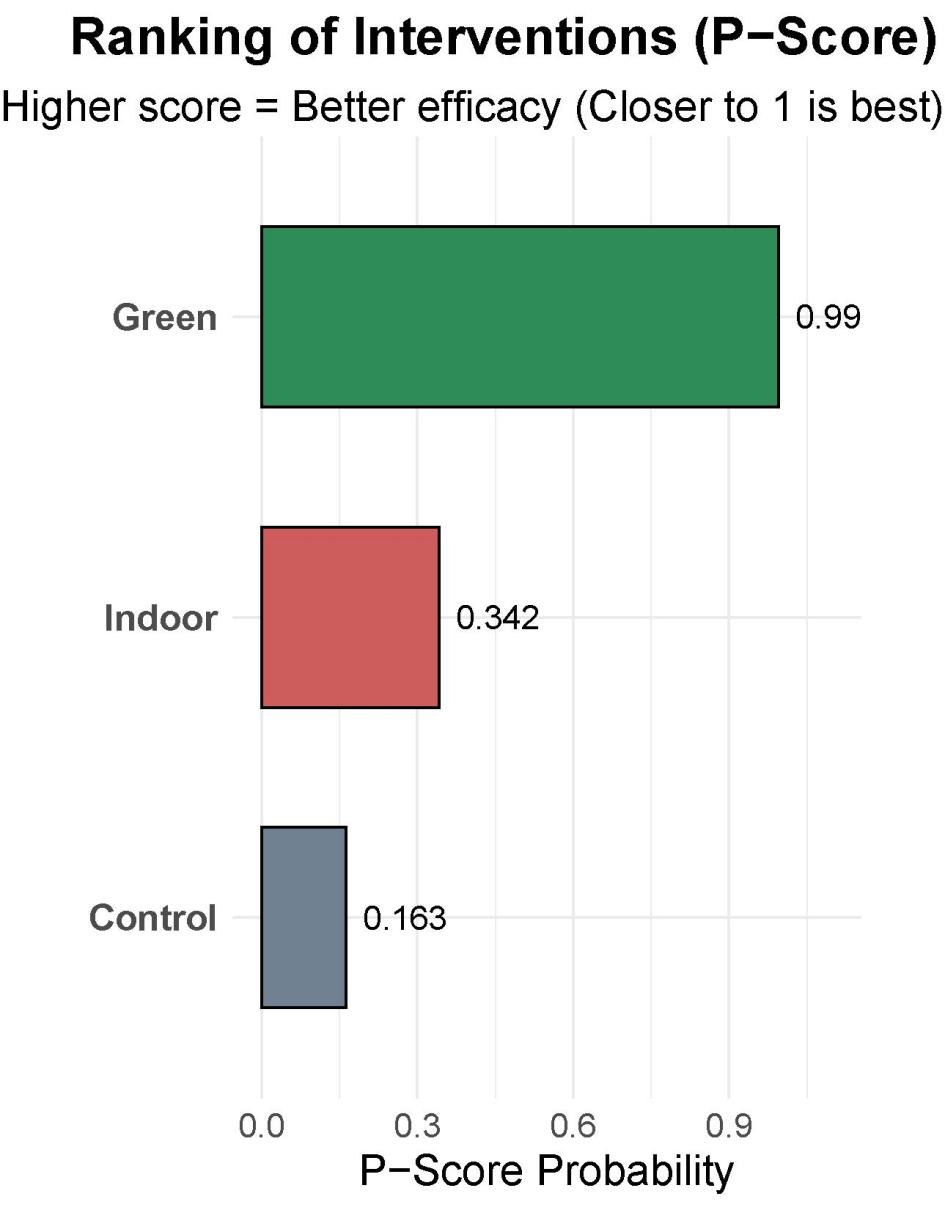
Figure S3. Ranking of interventions based on P-scores. P-scores measure the probability that an intervention is better than the competing interventions. A higher P-score (closer to 1) indicates a higher probability of being the most effective treatment.

Green: 0.99

Indoor: 0.342

Control: 0.163


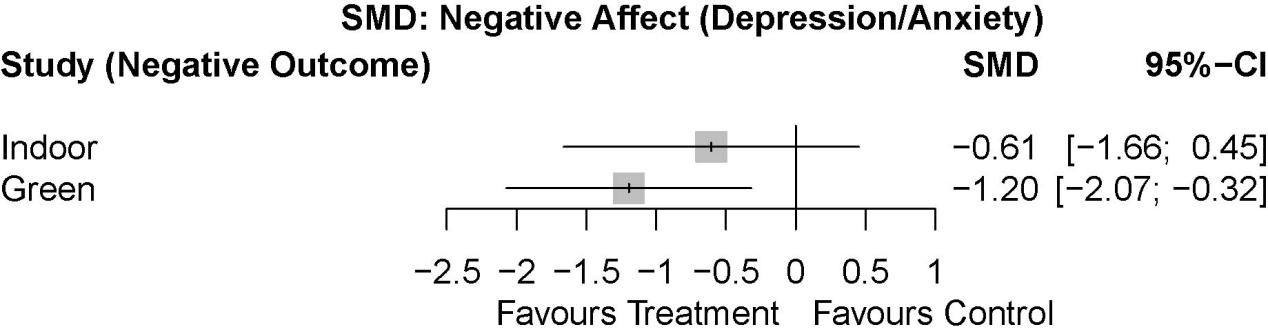


Figure S4. Subgroup analysis for negative affect outcomes. Comparison of Green and Indoor exercise versus Control on negative mental health indicators (e.g., depression, anxiety). Note: Values < 0 indicate improvement.


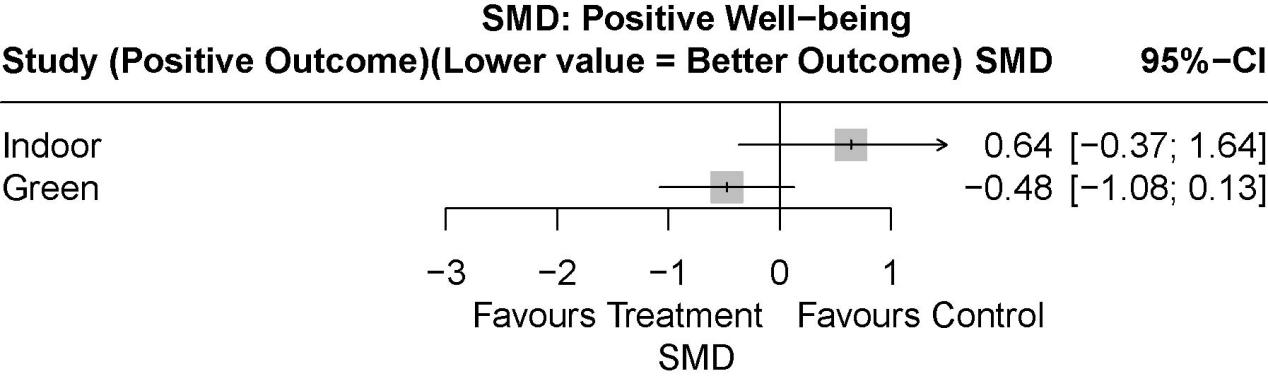


Figure S5. Subgroup analysis for positive well-being outcomes. Comparison of Green and Indoor exercise versus Control on positive mental health indicators (e.g., vitality, quality of life). Note: Positive scores were inverted so that values < 0 indicate improvement.


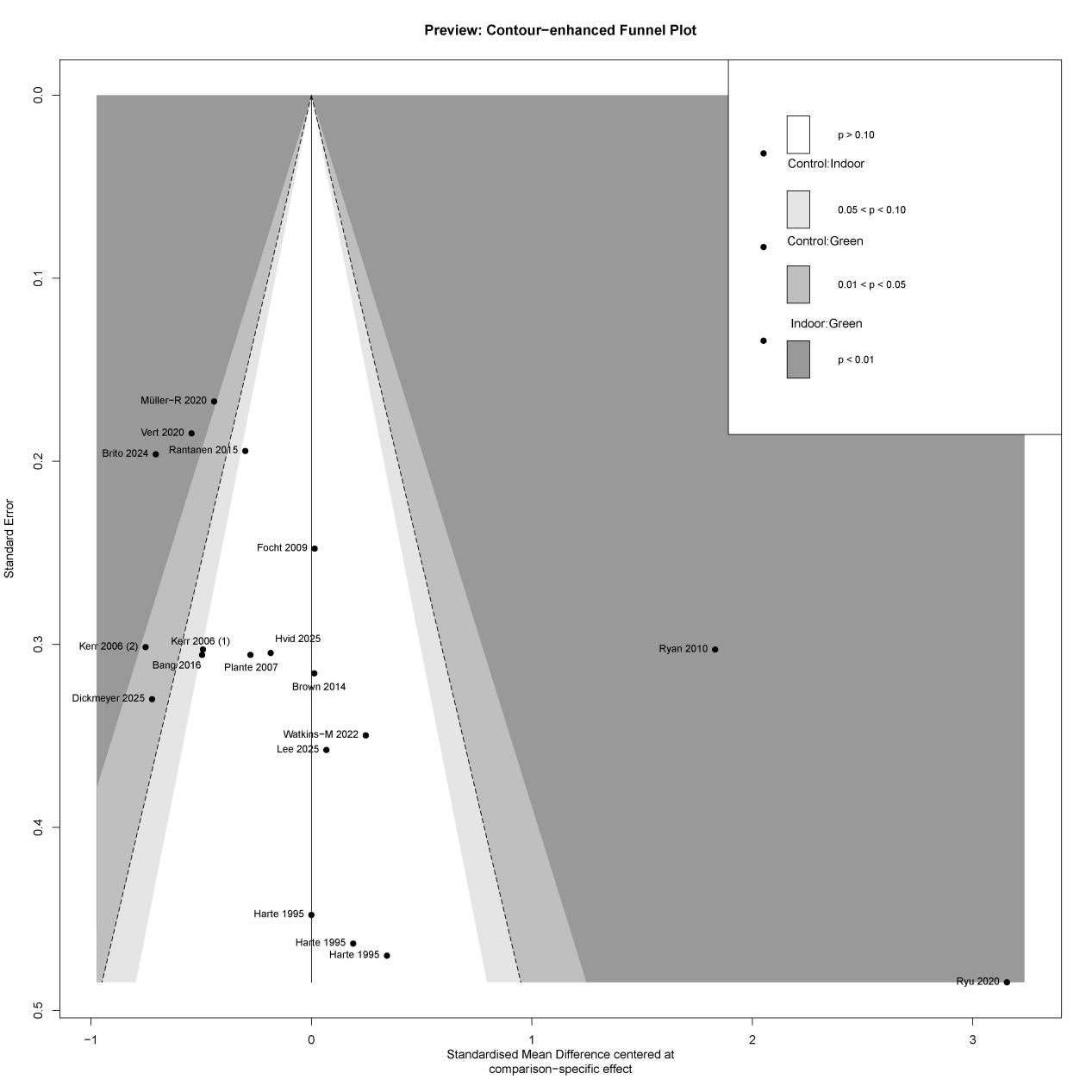
Figure S6. Contour-enhanced funnel plot for the evaluation of publication bias. The plot displays the SMD against the standard error. The shaded regions correspond to significance levels: white (p > 0.10), gray (0.05 < p < 0.10), and dark gray (p < 0.01). The symmetrical distribution suggests no substantial publication bias.
