# Supplementary material for: Comparative efficacy of green exercise versus indoor exercise for depression and anxiety: a systematic review and network meta-analysis
Source: Front Public Health. 2026 May 28;14:1831073. doi: 10.3389/fpubh.2026.1831073 (PMC13253268; doi:10.3389/fpubh.2026.1831073)
Supplement: Supplementary file 2 [file Table_1.docx]

| Category | Specifications & Keywords |
| --- | --- |
| Databases Searched | PubMed, Embase, Web of Science, and the Cochrane Central Register of Controlled Trials (CENTRAL). |
| Search Timeframe | From database inception to December 2025. (Note: The abstract mentions December 2024, while the Methods section specifies December 2025). |
| Concept 1: Population / Outcomes | depression, "anxiety", "mental health" |
| Concept 2: Interventions / Comparators | green exercise, "outdoor activity", "nature", "indoor exercise", "treadmill" |
| Concept 3: Study Design | randomized controlled trial |
| Limits & Restrictions | • Included: Adult participants (aged 18 years or older). <br>• Excluded: Studies involving virtual reality (VR) or simulated nature environments. |
| Supplementary Search Methods | Manual screening of the reference lists of included studies and relevant systematic reviews (snowballing). |

Table S1: Literature Search Strategy
